# Coronin 3 promotes gastric cancer metastasis via the up-regulation of MMP-9 and cathepsin K

**DOI:** 10.1186/1476-4598-11-67

**Published:** 2012-09-14

**Authors:** Gui Ren, Qifei Tian, Yanxin An, Bin Feng, Yuanyuan Lu, Jie Liang, Kai Li, Yulong Shang, Yongzhan Nie, Xin Wang, Daiming Fan

**Affiliations:** 1 State Key Laboratory of Cancer Biology and Xijing Hospital of Digestive Diseases, Xijing Hospital, Fourth Military Medical University, Xi’an, 710032, China

**Keywords:** Coronin 3, Gastric cancer, Metastasis, MMP-9, Cathepsin K

## Abstract

**Background:**

Coronins are a family of highly evolutionary conserved proteins reportedly involved in the regulation of actin cytoskeletal dynamics, although only coronin 3 has been shown to be related to cancer cell migration. In glioblastoma cells, the knockdown of coronin 3 inhibits cell proliferation and invasion. Coronin 3 is also associated with the aggression and metastasis of hepatocellular carcinoma. In this paper, we analyze the migration, invasion and metastasis abilities of gastric cancer cells after up- or down-regulation of coronin 3, and explore the mechanism of coronin 3 in the process of gastric cancer metastasis.

**Results:**

The expression of coronin 3 was higher in the highly metastatic sub-cell line MKN28-M, which we established in our laboratory. We also demonstrated that the expression of coronin 3 was remarkably higher in lymph lode metastases than in primary gastric cancer tissues, and over-expression of coronin 3 was correlated with the increased clinical stage and lymph lode metastasis. Recombinant lentiviral vectors encoding shRNAs were designed to down-regulate coronin 3 expression in gastric cancer cell lines. Stable knockdown of coronin 3 by this lentiviral vector could efficiently inhibit the migration and invasion of MKN45 gastric cancer cells. In contrast, up-regulation of coronin 3 significantly enhanced migration and invasion of MKN28-NM cells. In addition, knockdown of coronin 3 significantly reduced liver metastasis in mice after tail vein injection of gastric cancer cells. The Human Tumor Metastasis PCR Array was used to screen the metastasis-associated genes identified by the down-regulation of coronin 3, and the results suggested that, following the knockdown of coronin 3, the tumor cell migration and invasion were inhibited by the reduced expression of MMP-9 and cathepsin K.

**Conclusion:**

Coronin 3 is highly expressed in gastric cancer metastases and can promote the metastatic behaviors of gastric cancer cells, including their migration and invasion.

## Background

Cancer is characterized by proliferation, invasion, and metastasis, and more than 90% of mortalities are caused by metastases [1]. The first steps of metastasis include cancer cell adhesion, degradation of the ECM, and permeation of the basement membrane [2]. In most cases, cancer cell invasion during this process is dependent on the dynamic re-organization of the actin cytoskeleton, the dysfunction of cell adhesion, and the formation of invadopodia. Invadopodia are actin-rich membrane protrusions that are formed by invasive cancer cells, and they have a matrix-degradation activity that requires the polymerization and depolymerization of actin filaments [3]. For decades, the molecules and mechanisms involved in the regulation of the actin cytoskeleton have been an area of intense study [4]. There are a large variety of actin nucleators in human cells, such as the Arp2/3 complex, N-WASP, cortactin, cofilin, and coronins [5,6]. Studies evaluating the roles of the actin nucleation factors involved in cancer cell function may ultimately provide new treatments for invasive and metastatic cancers [7].

Coronins are highly conserved regulators of the actin cytoskeleton, and their structure and biological function have recently been described in detail [6]. Coronins bind F-actin as well as the Arp2/3 complex and are involved in inhibiting actin dynamics. Coronin 3 is a type I coronin protein with a calculated molecular mass of 53 kDa and is part of a conserved family of WD-repeat-containing, actin-binding proteins [8]. Similar to coronin1A and coronin1B, coronin 3 interacts with Arp2/3 and plays a negative role in actin polymerization. Additionally, recent studies have demonstrated that this protein participates in the metastatic behavior of many malignancies, such as diffuse glioma and primary effusion lymphoma [9,10]. Coronin 3 is also a novel biomarker for the invasive progression of hepatocellular carcinoma [11].

The present study conducted a more extensive investigation of the distribution of coronin 3 within gastric cancer tissues and related lymph lode metastases and demonstrated the potential of coronin 3 for use as a biomarker of cancer metastasis. The knockdown of coronin 3 expression in the MKN45 gastric cancer cell lines clearly decreased the migratory and invasion capabilities of these cells, while up-regulation of coronin 3 significantly enhanced migration and invasion of MKN28-NM cells. In addition, a tail vein metastasis assay showed that knockdown of coronin 3 significantly reduced liver metastasis of gastric cancer cells. The Human Tumor Metastasis PCR Array was then used to screen the metastasis-associated genes identified following the down-regulation of coronin 3, and this experiment revealed that nine of the 84 genes were down-regulated, whereas only 2 genes were up-regulated. Furthermore, we confirmed that the expression of MMP-9 and cathepsin K was positively correlated with the expression of coronin 3. In conclusion, these results suggest that coronin 3 may promote the invasion and metastasis of gastric cancer both *in vitro* and *in vivo* by regulating the expression of MMP-9 and cathepsin K.

## Results

### Coronin 3 expression is up-regulated in highly metastatic gastric cancers

**Figure 1:**
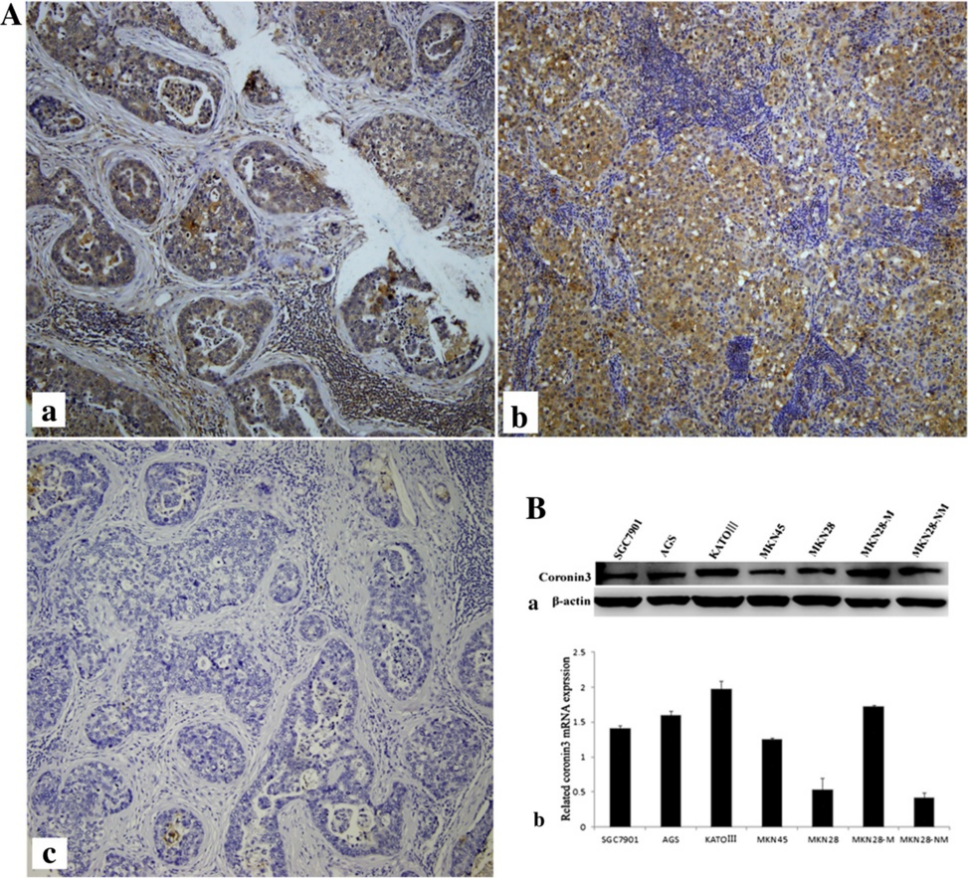
**The expression of coronin 3 in gastric cancer tissues and cell lines.A**. Immunohistochemical analysis of coronin 3 expression in gastric cancer and lymph lode metastasis tissues. **a**, primary gastric cancer tissues; **b**, lymph lode metastasis tissue; **c**, serial section with primary gastric cancer tissue (**a**) from the tissue array, negative control (pre-immune serum staining). **B**. Expression of coronin 3 in gastric cancer cell lines. β-actin was used as the internal control. The level of coronin 3 protein expression was significantly higher in the highly invasive MKN28-M cell subline than in the less invasive cell subline MKN28-NM. The level of coronin 3 mRNA expression was consistent with that of Coronin 3 protein expression.

Coronin 3 expression was examined in gastric cancer tissues and cell lines by first comparing coronin 3 expression in primary gastric cancer tissues and the related lymph lodes. A tissue array containing 40 gastric cancer and related metastatic lymph lode tissues was purchased from Aomei, China, and another 12 pairs of tissue samples were obtained from archives of Department of Pathology in Xijing Hospital between 2010 and 2011. The immunohistochemical results showed that coronin 3 was predominantly expressed in the cytoplasm of gastric cancer cells (Figure 1A). As shown in Table 1, ten primary gastric cancer tissue samples (19.2%) showed negative staining (0), whereas the staining in 17 (32.6%), 19 (36.5%), and 8 (15.3%) samples was scored as weakly positive (I), moderately positive (II), and strongly positive (III), respectively. For the related metastatic lymph lode tissues, the staining from 5 (9.6%), 7 (13.4%), 22 (42.3%), and 18 (34.6%) of the samples was scored as negative (0), weakly positive (I), moderately positive (II), and strongly positive (III), respectively. Therefore, coronin 3 expression was significantly higher in the metastatic lymph lode samples than in the primary cancer tissue samples (*P* = 0.03).

**Table 1:** Expression of coronin 3 in gastric cancer and related lymph node metastasis

**Histological type**	**n**	**Coronin 3 score (n)**				***P***
		**0**	**I**	**II**	**III**	
Primary cancer tissues	52	10	17	19	8	0.030*
Lymph node metastasis	52	5	7	22	18	

χ^2^ test were used to evaluate the significance of differences in two groups.*, *P* < 0.05.

The relationship between coronin 3 expression and clinicopathological parameters was analyzed. Coronin 3 expression in 152 gastric cancer tissue spots were detected by immunohistochemistry. Four spots were excluded because all fields were no cancer tissues but only fibrous or inflammatory tissues. As shown Table 2, over-expression of coronin 3 was correlated with the increased clinical stage (*P* = 0.001) and lymph lode metastasis (*P* < 0.001), but not statistically related to cancer differentiation, or patients’ gender and age.

**Table 2:** Clinic pathological association of coronin 3 in gastric cancer

**Category**	**n**	**Coronin 3 score (n)**				**p**
		**0**	**I**	**II**	**III**	
Gender						0.155
Male	99	13	22	28	36	
Female	49	11	10	18	10	
Age						0.995
≤58	85	14	18	27	26	
>58	63	10	14	19	20	
Differentiation						0.271
Well	13	4	4	3	2	
Moderately	34	7	9	11	7	
Poorly	101	13	19	32	37	
Stages						0.001*
I	22	6	7	6	3	
II	77	13	16	33	15	
III	35	3	7	6	19	
IV	14	2	2	1	9	
Lymph node metastases						<0.001*
0	100	18	28	37	17	
≥1	48	6	4	9	29	

The Kruskal–Wallis H-test and the Mann–Whitney *U* test were used to analyze the relationship between coronin 3 expression and clinicopathological factors. *, *P* < 0.05.

As shown in Figure 1B, coronin 3 expression was significantly elevated in SGC7901, AGS, KATOIII, and MKN45 cells, particularly in the highly invasive MKN28-M cell subline. This result was consistent with the mRNA expression levels from the qPCR analysis and suggested that the expression of coronin 3 is correlated with gastric cancer invasion.

### Coronin 3 is expressed in the cytoplasm of gastric cancer cells

**Figure 2:**
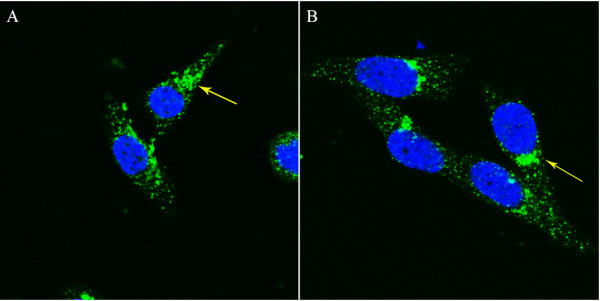
Coronin 3 was primarily localized in the cytoplasm of the MKN45 (A) and SGC7901 (B) cells (localization is marked by arrowheads).

As shown in Figure 2, the subcellular localization of coronin 3 in the SGC7901 and MKN45 gastric cancer cell lines was detected using a laser scanning confocal microscope. Coronin 3 expression was predominantly localized in the cytoplasm and surrounding the nucleus, and this finding is consistent with that of a previous study [12].

### Coronin 3 promoted the migratory, invasive, and *in vivo* metastatic abilities of gastric cancer cells

**Figure 3:**
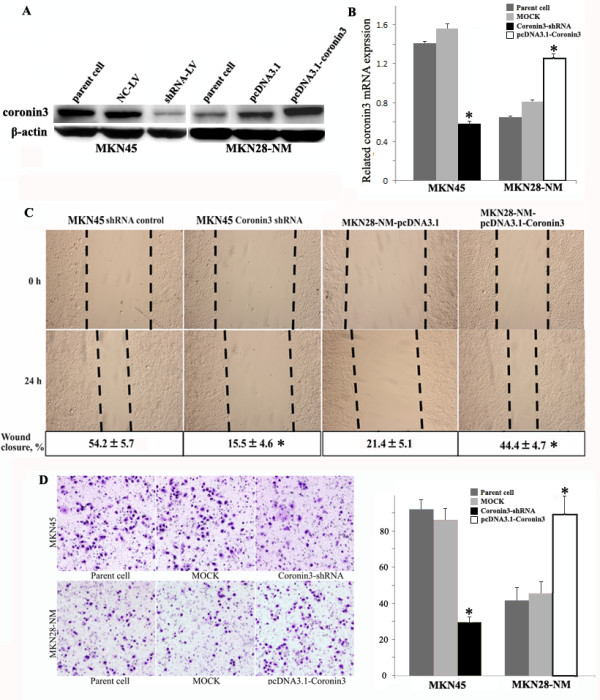
**The effects of Coronin 3 on the migration and invasion of gastric cancer cells.** The representative results of three similar experiments are shown. **A** and **B**. The MKN45 cells were infected with shRNA-LV, and the MKN28-NM cells were stably transfected with pcDNA3.1-Coronin 3. The protein and mRNA expression of Coronin 3 were then evaluated by Western blotting and qPCR. β-actin was used as the internal control. The MOCK samples were treated with scrambled shRNA-LV or the pcDNA3.1 vector. **C**. The migratory ability of the cells was evaluated with a wound-healing assay. The wound widths were measured at time 0 and 24 h after wounding, and the closure ratio was calculated in accordance with the following formula: wound closure (%) = (width 0 h) - width 24 h) / width 0 h. * p < 0.05. These results were then compared to those of the control cells. **D**. The invasive ability was evaluated by counting the number of cells that had invaded the Matrigel and the 8-μm-pore Transwell membrane. *, p < 0.05, compared to the cells infected with scrambled shRNA-LV or transfected with the pcDNA3.1 vector.

**Figure 4:**
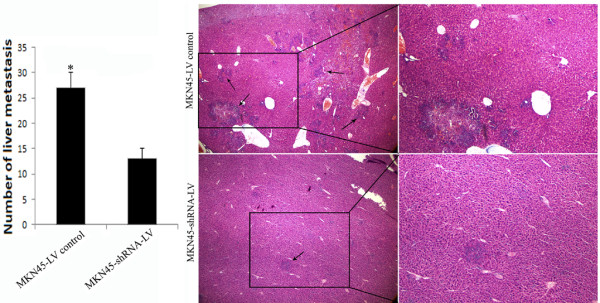
**Mice were tail vein-injected with 1x106 MKN45 cells infected with shRNA-LV or the control vector.** Each group contained 10 mice. The mice were sacrificed four weeks later, and the number of visible tumors in the liver was counted without magnification. The liver tissues were sectioned serially and then stained with H&E. *, p < 0.01. Liver metastases were marked by arrowheads.

To determine the role of coronin 3 expression in the malignant behavior of gastric cancer cells, a lentivirus containing an shRNA construct (shRNA-LV) was constructed to down-regulate coronin 3 expression in MKN45 cells, and pcDNA3.1-Coronin 3 was transfected to MKN28-NM cells to up-regulate coronin 3 expression. The mRNA and protein levels were determined by qPCR and Western blotting after infection or transfection. As shown in Figure 3A and 3B, coronin 3 expression was clearly down-regulated and had decreased by more than 85% in the MKN45 cells, and up- regulated by more than 60% in the MKN28-NM cells. This coronin 3 down-regulation or up-regulation showed no effect on the gastric cancer cell growth, and there were no differences between the MKN45-shRNA-LV or MKN28-coronin 3 cells and the control cells regarding levels of apoptosis or cell cycle progression, as assessed by flow cytometry (data not shown). We next evaluated the effect of coronin 3 expression on the invasive and migratory abilities of gastric cancer cells using an *in vitro* wound-healing assay and invasion assay. As shown in Figure 3C and 3D, the down-regulation of coronin 3 resulted in the marked inhibition of the migration abilities of MKN45 cells. Similar results were observed in the *in vitro* invasion assay. In contrast, up-regulation of coronin 3 significantly enhanced migration and invasion of MKN28-NM cells. In addition, a tail vein metastasis assay was used to examine the effects of coronin 3 down-regulation on the *in vivo* metastasis of MKN45 cells. MKN45-shRNA-LV, or control cells (1 × 10^6^) were injected into the tail veins of SCID mice. The extent of the metastatic tumors on the surface of the liver was significantly reduced in mice that received treated cells compared to those that received control cells (Figure 4, P < 0.05). Thus, the results of both the *in vitro* and *in vivo* assays suggest that coronin 3 plays a significant role in gastric cancer metastasis.

**Figure 5:**
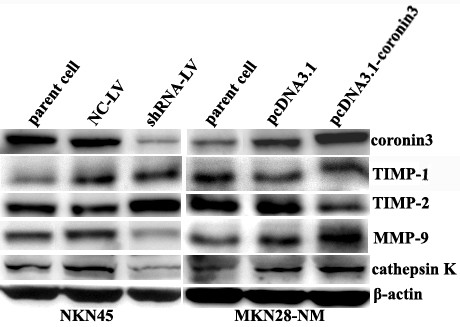
**The effect of Coronin 3 expression on several proteolytic enzymes in gastric cancer cells.** The expression of TIMP-2, MMP-9, and cathepsin K in gastric cancer cells was evaluated by Western blotting. β-actin was used as the internal control. The levels of TIMP-2, MMP-9, and cathepsin K protein in the MKN45-shRNA-LV and MKN28-pcDNA3.1-Coronin 3 cells were significantly different from those in the empty vector-transfected cells (p < 0.05).

### Real-time PCR array analysis of coronin 3-regulated metastasis-related gene expression profiles

Numerous molecules are involved in the migration and invasion of gastric cancers. In this study, we compared the gene expression patterns of cells following the down-regulation of coronin 3 with those of untreated control cells. Total mRNA from MKN45-shRNA-LV cells and controls was harvested and subsequently screened against a library of 84 metastasis-related genes. Genes with more than a twofold increase or decrease in expression were considered significantly regulated by coronin 3. Nine of the 84 genes were down-regulated, whereas only 2 genes were up-regulated (Table 3). However, only 2 of these genes, MMP-9 and CTSK (cathepsin K), were verified by the results of the qPCR, and Western blotting.

**Table 3:** mRNA expression of metastasis-related genes after down-regulation of coronin 3

**Gene name**	**Description**	**Fold Up- or Down-Regulation**
CDH11	Cadherin 11, type 2, OB-cadherin (osteoblast)	−2.59
CDH6	Cadherin 6, type 2, K-cadherin (fetal kidney)	−6.62
CTSK	Cathepsin K	−11.78
CTSL1	Cathepsin L1	12.08
CXCL12	Chemokine (C-X-C motif) ligand 12	2.08
CXCR2	Chemokine (C-X-C motif) receptor 2	−3.08
ITGA7	Integrin, alpha 7	−3.53
MMP9	Matrix metallopeptidase 9	−2.03
SMAD4	SMAD family member 4	−2.06
TIMP3	TIMP metallopeptidase inhibitor 3	−7.16
VEGFA	Vascular endothelial growth factor A	−3.19

Proteases are known to play causal roles in the malignant progression of human tumors [13]. The initial step of the metastatic process involves the degradation of the basement membrane and extracellular matrix (ECM) by various types of proteases, such as MMPs, TIMPs, and serine and cysteine proteases [14,15]. Therefore, we examined the effect of coronin 3 knockdown or up-regulation on the expression of MMP-9, cathepsin K (based on the results of the PCR array), MMP-2, TIMP-1 and TIMP-2 in gastric cancer after infection. These data confirmed that the expression of MMP-9 and cathepsin K was down-regulated due to the coronin 3 knockdown in the MKN45 cells and up-regulated when MKN28-NM cells were transfected with pcDNA3.1-Coronin 3. The result of TIMP-2 was on the contrary. However, the expression of TIMP-1 was not altered due to coronin 3 knockdown. For these experiments, the β-actin expression level was used as an internal control (n). Taken together, these results suggest that MMP-9, TIMP-2 and cathepsin K at least partially contribute to coronin 3-mediated gastric cancer metastasis.

## Discussion

Cancer metastasis is the main cause of all cancer-related deaths [16]. Metastasis is a multistep process that is referred to as the invasion-metastasis cascade, and the first critical step of this process is the invasion of primary cancer cells into the surrounding ECM and stromal cell layers [17]. Reassembly of the actin cell skeleton, aberrant cell-cell contact, and protrusion formation are indispensable components of this step of metastasis for almost all epithelial cancers [18].

Many regulatory proteins are required for cell migration and possess actin polymerization- and depolymerization-regulating effects. Of these effects, the actin-related protein (Arp) 2/3 complex plays a vital role in actin polymerization and its regulation [19]. Several nucleating promoting factors (NPF), particularly N-WASP, activate the Arp2/3 complex and then initiate the formation of angular actin filament branches on the sides of preexisting filaments [20]. In contrast, the coronin family can bind to and inhibit Arp2/3 complex activity. Cai et al. reported that coronins can limit Arp2/3-dependent actin branching by inhibiting Arp2/3 docking or by facilitating debranching. Moreover, this function is antagonized by cortactin, which is an additional factor that can bind to the Arp2/3 complex and synergistically activate the complex [21,22]. Together, the interaction of NPFs and coronins with the Arp2/3 complex coordinates actin polymerization and depolymerization to mediate cell migration and invasion. Coronin 3 has a structure similar to that of other coronins and can bind to Arp2/3 and F-actin, although the functional significance of this interaction remains unclear [23]. However, coronin 3 is the only member of this family that has been associated with the metastasis of neoplasms, such as diffuse glioma, primary effusion lymphoma and hepatocellular carcinoma [24-26].

In this study, we first examined the expression of coronin 3 in primary gastric cancer tissues and lymph lode metastases. These results demonstrated that coronin 3 was more highly expressed in lymph lode metastases as compared to primary tissues, and this expression was also greater in MKN28-M cells compared to MKN28-NM cells. These data suggest that elevated expression of coronin 3 is associated with the high metastatic potential of gastric cancers and that coronin 3 may participate in the process of invasion and metastasis to play a positive role in the metastasis of gastric cancer. To confirm this hypothesis by immunohistochemistry and Western blotting, we observed the migration and invasion ability of gastric cancer cell lines after knocking down or up-regulating the expression of coronin 3 with a shRNA-LV vector or pcDNA3.1-Coronin 3, respectively. These experiments demonstrated that coronin 3 could significantly promote the invasion and metastasis of gastric cancer cells.

In addition, we explored the mechanism behind the association between coronin 3 and gastric cancer metastasis and identified downstream effectors contributing to this process. We used the Human Tumor Metastasis PCR Array to confirm that two proteases (MMP-9 and cathepsin K) were responsible for coronin 3-related metastasis. Invadopodia formation and ECM degradation have been shown to be associated with cell invasion, and for these processes, certain proteases that are responsible for ECM degradation are also regulated by proteins that participate in actin reassembly, such as members of the WASP/WAVE family and cortactin [27-29]. In addition, MMPs may be required for the initial steps of invadopodia formation [30]. In our study, we found that the expression of MMP-9 and cathepsin K showed significant positive correlation with coronin 3 expression, whereas TIMP-2 expression shows the opposite results. K. Sossey-Alaoui et al. [31] reported that WASP/WAVE family (a kind of NPF) could regulate of MMPs expression and activity via the p38 pathway. As described above, coronins family interact with NPF and other actin polymerization- and depolymerization-regulating protein. Therefore, Coronin 3 may regulate protease expression via MAPK pathway, which needs further exploration.

## Conclusion

In the present study, we have provided clear evidence that coronin 3 is highly expressed in metastatic gastric cancer tissues and that coronin 3 can promote gastric cancer metastasis, at least in part by up-regulating cathepsin K and MMP9 expression and down-regulating TIMP2 expression.

## Methods

### Cell lines and gastric cancer tissues

The SGC7901, AGS, KATOIII, MKN-45, and MKN-28 human gastric cancer cell lines were maintained in our laboratory. Additionally, sublines with a low (MKN28-NM) and high degree of invasiveness (MKN28-M) were derived from the MKN28 parental cell line by our laboratory [32]. All of the cell lines were grown in RPMI 1640 supplemented with 10% fetal calf serum (FBS) and incubated at 37°C with 5% CO2 in a humidified incubator. Twelve gastric cancer tissues and lymph lode metastases were obtained from archives of Department of Pathology in Xijing Hospital between 2010 and 2011. Human ethics approval was duly obtained from the Xijing Hospital.

### Tissue array

Commercially available adult human cancer tissues and lymph lode metastases, in the form of formalin-fixed microarrays, were obtained from Aomei (Aomei Biotechnology Co. Ltd., Xi’an, China). One cancer tissue array (AM01C09) contained 80 spots, with 40 gastric adenocarcinoma spots and 40 matched lymph lode metastases spots from 40 patients (aged 24–74 years, 1.5-mm diameter per spot). Another tissue array (AM01C04) contained 152 gastric cancer spots from 152 patients (aged 17–84 years, 1mm diameter per spot), and the patients’ gender, age, and clinicopathological parameters were provided by the manufacturer.

### Immunohistochemistry

The coronin 3 immunostaining was performed on all of the tissues using the standard avidin–biotin peroxidase staining technique. Briefly, the paraffin-embedded tissues were deparaffinized in xylene and rehydrated in alcohol and were then incubated in 3% H_2_O_2_ for 10 min to block endogenous peroxidase activity. The samples were then incubated with normal goat serum (Zhong Shan Bio, China) for 30 min at room temperature. Subsequently, a mouse monoclonal antibody against coronin 3 (1:100 dilution) (Abcam, USA.) was applied to the tissue array and incubated overnight at 4°C, and preimmune sera were used instead of primary antibodies for the negative control samples. SP kits (Zhongshan Bio) were used according to the manufacturer’s instructions for detection. The peroxidase activity was visualized with the addition of DAB solution, and the samples were then counterstained with hematoxylin.

All tissues or spots were scored by two independent observers in a blinded fashion, and the observers did not have prior knowledge of the clinicopathological details of the samples. The coronin 3 expression was evaluated according to the fraction of positive cells per specimen and the staining intensity, which was evaluated using the following histological scoring method as previously described [33]. The fraction of positive cells per specimen was quantitatively evaluated and assigned a score of 0 for 0% staining of the cells examined, a score of 1 for 0.01 - 25% staining, a score of 2 for 25.01 - 50% staining, a score of 3 for 50.01 - 75% staining, or score of 4 for greater than 75% staining. The intensity was graded as follows: 0, no signal; 1, weak signal; 2, moderate staining signal; and 3, strong staining. The histological score for coronin 3 expression in each spot was computed using the following formula: histological score = ratio score × intensity score. A total score, ranging from 0–12, was determined and graded as either negative (0, score = 0–1), weak (I, score = 2–4), moderate (II, score = 5–8), or strong (III, score = 9–12) for use in further nonparametric tests.

### Western blotting and antibodies

The cell samples were homogenized in an SDS-PAGE sample buffer containing 2% SDS. The protein samples were prepared as previously described [34]. After the samples were heated to 100°C for 5 min and clarified by spinning in a microcentrifuge at top speed and at room temperature for 5 min, the total protein extracts were resolved in a polyacrylamide gel using the Laemmli buffer system. The gels were electrically transferred to nitrocellulose membranes at 20 V for 25 min. After blocking in Tris-buffered saline containing 10% non-fat-milk, the nitrocellulose membranes were incubated with specific antibodies against coronin 3 (Abcam, USA), cathepsin K, TIMP-1 (Santa Cruz, USA), TIMP-2, MMP-9 (Cell Signal Technology, USA). The membranes were washed with Tris-buffered saline containing 0.05% Tween-20 and were then incubated with peroxidase-conjugated anti-mouse or anti-goat IgG secondary antibodies (Zhong Shan Bio, China) and washed once more. The blots were developed using an ECL substrate solution (GE, USA).

### Total RNA extraction and real-time PCR

Total RNA from the SGC7901, AGS, KATOIII, MKN45, MKN28-NM and MKN28-M gastric cancer cell lines was extracted using Trizol (Invitrogen, Carlsbad, CA), and cDNA was synthesized using the PrimeScript® RT reagent kit (TaKaRa Biotechnology, Dalian, China), according to the manufacturer’s recommendations. A LightCycler FastStart DNA Master SYBR Green I System (Roche, Basel, Switzerland) was used for the real-time PCR, as previously described [35]. Briefly, the 25-μl reaction contained 12.5 μl of SYBR Green qPCR master mix (TaKaRa Biotechnology, Dalian, China), 10 nmol of each primer, 2.0 μl of the DNA template and 8.5 μl of dH_2_O. The PCR cycling conditions consisted of 40 cycles of the following steps: 95°C for 30 s, 58°C for 40 s, and 72°C for 30 s. β-actin mRNA was used as the internal control, and the reaction mix without the template DNA was used as the negative control. All of the samples were measured 3 times independently, and the resulting fluorescence curves represented the number of copies expressed. The following primer sequences were used: β-actin, 5'- ATA GCA CAG CCT GGA TAG CAA CGT AC-3' (forward) and 5'- CAC CTT CTA CAA TGA GCT GCG TGT G-3' (reverse); and coronin 3, 5'- CTG CAC AGC TTC CAA AGA CAA GA-3' (forward) and 5'- GGC TGA ACC CAG TGG TGA AGA -3' (reverse).

### Laser scanning confocal microscope analysis

The SGC7901 and MKN45 cells were seeded on cover slips and incubated in a humidified incubator and normal medium for 24 hours. The cover slips were fixed in 4% paraformaldehyde for 20 min and blocked in blocking solution (Beyotime, China) for 1 hour. The cover slips were incubated with mouse anti-coronin 3 monoclonal antibody (1:100 dilution) overnight at 4°C, and then incubated with FITC-conjugated AffiniPure donkey anti-mouse IgG (1:200; red fluorescence, Jackson Immunoresearch Laboratories, Inc.) as the secondary antibody for 1.5 h at room temperature. Then, DAPI (Beyotime) was applied for 5 min to stain the nucleus. The cover slips were mounted and detected under laser scanning confocal microscope (FV1000, Olympus).

### Transfection and Lentiviral transduction of shRNA vectors

The pcDNA3.1-Coronin 3 construct was a gift from Prof. CS Clemen at the Institute of Biochemistry I of Medical Faculty at the University of Cologne, Germany. The MKN28-NM cells were plated in six-well plates and cultured in RPMI1640 supplemented with 10% FBS. At 90% confluence, the cells were transfected with pcDNA3.1-Coronin 3 or a pcDNA3.1 empty vector (Mock) using Lipofectamine^TM^ 2000 (Invitrogen, USA). The expression level of coronin 3 was evaluated by Western blot analysis.

The shRNA-oligo 5^′^-CGU CCA CUA CCU CAA CAC AUU-3^′^[10] was used to down-regulate the expression of human coronin 3 (GeneBank, GI: NM_014325). The scrambled shRNA sequence served as the negative control. The oligo shRNA and negative control sequences were subcloned into pGCSIL-GFP, and shRNA plasmids were co-transfected with lentiviral packaging plasmids. The resulting lentivirus and negative control were then infected into the MKN45 cell lines, according to the manufacturer’s protocol (GeneChem, Shanghai, China). The infected cells were sorted by flow cytometry based on green fluorescent protein (GFP) expression.

### Wound-healing assay

For wound-healing assays, 2 × 10^6^ cells were seeded into the 60-mm-diameter dishes. Draw a line across the center of the plate with plastic pipette tip to produce a sharp 1-mm-wide wound area after the cells have reached confluency. After 48 h culturing in medium with 1% fetal bovine serum (FBS), cell movement into the wound area was examined using a phase-contrast microscope.

### Cell invasion assay

The cell invasion assays were performed (as previously described [36]) using Transwell plates (8 μm pore size, Corning Costar Corp.). Fifty microliters of Matrigel, which had been diluted to a concentration of 2 mg/mL, were added to the lower surface of a polycarbonate membrane and air-dried. The polycarbonate membrane was rinsed with PBS, the inserts were plated into the wells, and 500 μl of RPMI-1640 containing 10% FBS was added to the lower chamber. One hundred microliters of a freshly prepared MKN28-NM or MKN45 cell suspension (at 5 × 10^5^ cells/ml) in RPMI-1640 containing 1% FBS was then added to the upper chamber. The cells were incubated for 36 h at 37°C in a 5% CO_2_ humidified incubator and would invade to the lower chamber. After incubation, cells were scraped from the upper surface of the membrane with a cotton swab. and the invaded cells on the bottom surface were fixed with methanol and stained with crystal violet. The invasive capability was determined by the number of penetrating cells under a microscope on 10 random fields in each well.

### Tail vein metastatic assay

The tail vein metastatic assay was analyzed as previously described [36]. Nude mice were handled using best humane practices and were cared for in accordance with NIH Animal Care and Use Committee guidelines. Cells were harvested using trypsin and washed twice with PBS. One hundred microliter PBS containing 1 × 10^6^ cells were injected through tail vein, after which the overall health condition and body weight of the mice were monitored. Four weeks after the injection, the mice were sacrificed and the liver tissues were isolated. After counting the number of visible tumors on liver surface, the liver tissues were made into serial sections before HE staining and observed under a light microscope. There were 10 mice in each group.

### Tumor metastasis PCR array

The expression patterns of the metastasis-related genes were analyzed using the Human Tumor Metastasis PCR Array (APHS-028A; SuperArray Inc.), which was obtained from Kangchen Genechip in Shanghai, China. Total RNA was extracted from MKN45-shRNA-LV-treated cells and control cells using standard protocols and was then converted to first-strand cDNA. The cDNA template was combined with a 2× SuperArray PCR master mix, the mixture was added to the wells of the PCR Array plate (384-well) containing the gene-specific primer sets, and real-time PCR was performed. The PCR cycling conditions consisted of 40 cycles of 95°C for 15 s, 60°C for 1 min, and 72°C for 30 s. Five housekeeping genes were used as internal controls. The ΔCt value for each metastasis-related gene in each treatment group was then calculated. The differential expression of each gene was measured according to the comparative Ct method (ΔΔCt), which assessed the fold-change difference in comparison to the MKN45-shRNA-LV control expression as 2^-ΔΔCt^. If the fold-change was greater than 2, the result was reported as a fold up-regulation. If the fold-change was less than 2, the negative inverse of the result was reported as a fold down-regulation.

### Statistical analysis

The statistical analysis was performed using SPSS 17.0 software. χ^2^ tests were used to evaluate the significance of differences in coronin 3 expression frequency between gastric cancer tissues and lymph lode metastases. The Kruskal–Wallis H-test and the Mann–Whitney U test were used to analyze the relationship between coronin 3 expression and clinicopathological factors of gastric cancer samples. A one-way ANOVA was used for analyzing the results of the migration and invasion assays. Differences were considered statistically significant for *p* values less than 0.05.

## Competing interests

The authors have no financial or non-financial competing interest to declare.

## Authors’ contributions

GR, QT and YA carried out most of the experiments. BF and YL carried out statistical analysis. JL, YS discussed the design of the experiments, the results and assisted in writing. GR, YN, XW and DF designed the experiments and participated in writing the manuscript. All authors read and approved the final manuscript.
