# Correction: Coronin 3 promotes gastric cancer metastasis via the up-regulation of MMP-9 and cathepsin K

**DOI:** 10.1186/s12943-022-01674-x

**Published:** 2022-10-25

**Authors:** Gui Ren, Qifei Tian, Yanxin An, Bin Feng, Yuanyuan Lu, Jie Liang, Kai Li, Yulong Shang, Yongzhan Nie, Xin Wang, Daiming Fan

**Affiliations:** State Key Laboratory of Cancer Biology and Xijing Hospital of Digestive Diseases, Xijing Hospital, Fourth Military Medical University, Xi’an, 710032 China

**Correction: Mol Cancer 11, 67 (2012)**

**https://doi.org/10.1186/1476-4598-11-67**

In the originally published version of this article [1], Fig. 3D (the bottom row on the right, labeled as pcDNA3.1-Coronin3) was used incorrectly after checking the original records, and the correct pictures of the experimental group was replaced.

**Fig. 3:**
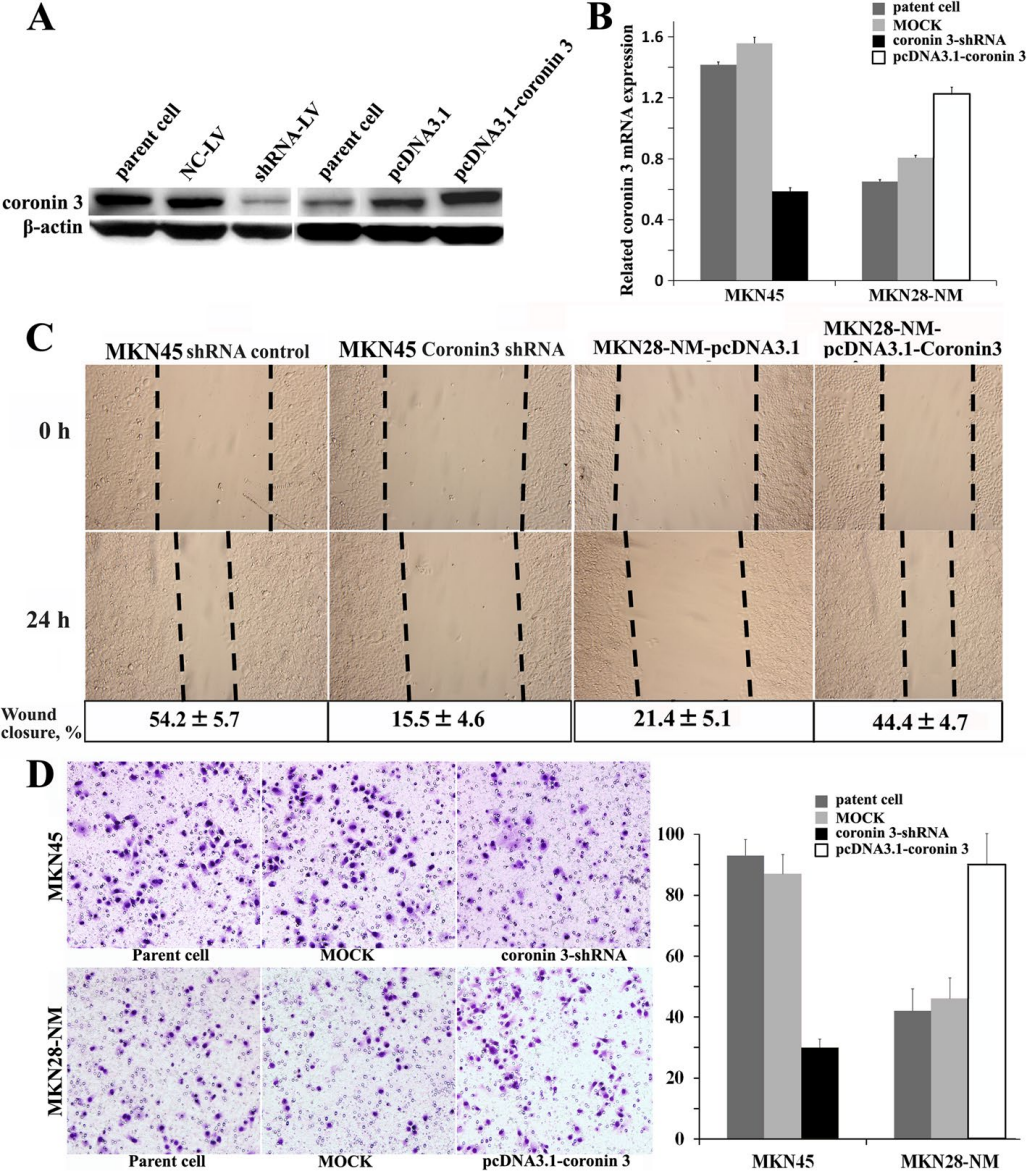
The effects of Coronin 3 on the migration and invasion of gastric cancer cells. The representative results of three similar experiments are shown. **A** and **B**. The MKN45 cells were infected with shRNA-LV, and the MKN28-NM cells were stably transfected with pcDNA3.1-Coronin 3. The protein and mRNA expression of Coronin 3 were then evaluated by Western blotting and qPCR. β-actin was used as the internal control. The MOCK samples were treated with scrambled shRNA-LV or the pcDNA3.1 vector. **C**. The migratory ability of the cells was evaluated with a wound-healing assay. The wound widths were measured at time 0 and 24 h after wounding, and the closure ratio was calculated in accordance with the following formula: wound closure (%) = (width 0 h) - width 24 h) / width 0 h. * *p* < 0.05. These results were then compared to those of the control cells. **D**. The invasive ability was evaluated by counting the number of cells that had invaded the Matrigel and the 8-μm-pore Transwell membrane. *, *p* < 0.05, compared to the cells infected with scrambled shRNA-LV or transfected with the pcDNA3.1 vector

## References

[1] Ren G, Tian Q, An Y (2012). Coronin 3 promotes gastric cancer metastasis via the up-regulation of MMP-9 and cathepsin K. Mol Cancer.

